# Family physicians have greater ambiguity tolerance in the clinical context: A nationwide cross‐sectional study

**DOI:** 10.1002/jgf2.747

**Published:** 2024-10-28

**Authors:** Hirohisa Fujikawa, Takuya Aoki, Takayuki Ando, Junji Haruta

**Affiliations:** ^1^ Center for General Medicine Education, School of Medicine Keio University Shinjuku‐ku Tokyo Japan; ^2^ Department of Medical Education Studies, International Research Center for Medical Education, Graduate School of Medicine The University of Tokyo Bunkyo‐ku Tokyo Japan; ^3^ Division of Clinical Epidemiology The Jikei University School of Medicine Minato‐ku Tokyo Japan; ^4^ Section of Clinical Epidemiology, Department of Community Medicine, Graduate School of Medicine Kyoto University Sakyo‐ku Kyoto Japan; ^5^ Medical Education Center, School of Medicine Keio University Shinjuku‐ku Tokyo Japan

**Keywords:** ambiguity tolerance, clinical context, family physicians, tolerance for ambiguity, tolerance of ambiguity

## Abstract

**Background:**

Ambiguity tolerance in the clinical context is increasingly recognized as essential for physicians to work as professionals. However, the relationship between specialty and ambiguity tolerance in the clinical context has been understudied. Here, we investigated the association between specialty and ambiguity tolerance in the clinical context, focusing on differences between family physicians (FPs) and non‐FPs.

**Methods:**

We performed a nationwide cross‐sectional study in Japan. We asked FPs from 14 family medicine residency programs across Japan and non‐FPs from monitors of an internet survey company in Japan to participate in the study. We assessed their tolerance for ambiguity using the Japanese version of the Tolerance for Ambiguity in Medical Students and Doctors (J‐TAMSAD) scale.

**Results:**

In total, 388 physicians (178 FPs and 210 non‐FPs) completed our anonymous online survey and were included in the analysis. After adjustment for possible confounders (gender and postgraduate years), FPs had higher J‐TAMSAD scale scores than internists/pediatricians, surgeons, and physicians with other specialties, meaning that FPs had greater ambiguity tolerance.

**Conclusions:**

This study reveals that FPs had greater tolerance for ambiguity in the clinical context than non‐FPs. Our findings suggest that there may be a need to increase non‐FP's tolerance for ambiguity specific to the clinical context through educational interventions, since ambiguity is inherent and growing in medicine today. FPs and non‐FPs should work together to complement each other's strengths, rather than simply improving the training of non‐FPs.

## 1 INTRODUCTION

Tolerance for ambiguity is increasingly recognized as a crucial competency in the context of professional medical care.^1^, ^2^ Healthcare professionals and students worldwide are experiencing an increase in ambiguity.^3^ Ambiguity is defined as a lack of reliable, credible, or adequate information, and tolerance to it is the tendency to perceive situations that are ambiguous as being desirable.^4^ Ambiguity tolerance can affect the ethical behavior of physicians and the quality of their patient care. Wayne et al.^5^ showed that medical trainees with lower tolerance for ambiguity might be more likely to avoid underserved patients. Lower tolerance of ambiguity is associated with a greater need for cognitive closure^6^ and may therefore lead to increased fear of making mistakes,^7^ increased test‐ordering behavior,^8^, ^9^ and engaging in defensive medical practice.^10^ Ambiguity tolerance may have a positive association with physicians' empathy.^2^, ^11^ Since ambiguity is inherent in practicing medicine,^12^ a better understanding of tolerance for ambiguity among physicians is of high priority in the education of health professionals.

Ambiguity tolerance is associated with individual characteristics. Prior studies have indicated associations between tolerance for ambiguity and stage of training, and between tolerance for ambiguity and gender.^13^, ^14^ Leung et al.^15^ showed a link between a personality profile and ambiguity tolerance among medical trainees. A recent study in Canada showed that medical trainees with a history of individual sport had greater tolerance of ambiguity than those with a history of team sport.^16^

Conversely, there are individual factors in tolerance that have not yet been considered, such as specialty. Every specialty has some degree of ambiguity and is expected to tolerate and manage it. However, the finding of a significant relationship between physician specialty and ambiguity tolerance would provide crucial insights for educators and residency program directors in tailoring educational programs that specifically enhance and address the capacity for ambiguity tolerance.

In particular, because family physicians (FPs) care for patients with a wide range of problems,^17^ see patients with undifferentiated chronic and acute conditions,^18^ and work in locations with limited access to testing, they have been considered more likely to encounter ambiguous situations than other specialties and to have high ambiguity tolerance.^17^, ^19^ Contrary to this expectation, however, Geller et al.^20^ showed that psychiatrists' tolerance for ambiguity was higher than that of other specialties, and that family practitioners had the lowest tolerance. This study was limited in two aspects, however, and the finding warrants re‐examination. First, the authors did not measure ambiguity tolerance specific to the clinical context. Although the classification of ambiguity tolerance has not been determined, it can be divided into two classifications: ambiguity tolerance as a personality trait and ambiguity tolerance specific to the clinical context. Whereas the former is challenging to alter through experience, the latter may be modifiable.^21^ Consequently, assessing ambiguity tolerance necessitates a scale that is specifically designed for the clinical setting. Second, the study was conducted in the early 1990's. The medical profession has undergone significant changes over the past few decades, including changes in medical students' preferences for specialization.^22^ Accordingly, it is important that the link between specialty and ambiguity tolerance be verified in the context of present‐day medicine.

Most of the previous studies of specialty and ambiguity tolerance focused on undergraduate medical students. For example, Babenko et al.^23^ elucidated the association between tolerance for ambiguity and prospective specialty choice among students in Canada. They found no significant difference in ambiguity tolerance between students interested in family medicine and those interested in other specialties.^23^ Conversely, there is a paucity of studies that have compared ambiguity tolerance between specialties in which medical graduates have actually progressed.

Here, we aimed to examine the association between specialty and ambiguity tolerance specific to the clinical context with a focus on differences between FPs and non‐FPs.

## 2 METHODS

### 2.1 Study design

This study is part of a series of studies on ambiguity tolerance and factors related to it among Japanese physicians. It is based on a nationwide cross‐sectional online survey in February 2024.

### 2.2 Study participants

This study involved physicians across Japan. In the Japanese postgraduate medical education system, postgraduate year (PGY) 1–2 physicians (junior residents) rotate through several clinical departments on a monthly basis. After 2 years of training, physicians proceed to advanced clinical training in their selected specialty, which usually lasts for 3–4 years (senior residents). Accordingly, we excluded junior residents and included physicians at or above PGY 3.

In Japan, FPs are known by various names (e.g., general practitioners and general medicine physicians, in addition to FPs).^24^, ^25^ In this study, we defined FPs as “Japan Primary Care Association (JPCA) certified primary care physicians, JPCA certified FPs, general medicine specialists, or trainees in these specialties.” The JPCA was established in 2010 and is the recognized certifying body for primary care physicians.^26^ The training system of JPCA‐certified FPs has obtained international certification from the World Organization of National Colleges, Academies and Academic Associations of General Practitioners/Family Physicians.^27^ General medicine specialists are accredited by the Japanese Medical Specialty Board.^28^ Common to all is that they provide comprehensive care and perform a wide range of tasks to respond flexibly to the needs of their respective clinical settings.^24^, ^25^ Therefore, since they are likely to encounter ambiguous situations, we decided to include them in the study as FP participants.

We asked a survey company (X company) to recruit physicians in specialties other than family medicine. These were all registered monitors of X company. Among the monitors, those specializing in family medicine were excluded, and those remaining were randomly selected and invited to participate in our study by X company. Conversely, to recruit FPs, since the researchers are all FPs, we asked the directors of 14 family medicine residency programs across Japan to distribute our questionnaire in their programs. We chose this strategy to recruit FPs for two reasons. First, FPs currently represent a small percentage (less than 1%) of physicians in Japan,^29^ and few FPs were registered by the survey company. Second, to obtain a sample of FPs that was as large in number and with as high a response rate as possible.

### 2.3 Data collection

Participants were asked to complete an online self‐administered anonymous questionnaire. While surveys for FPs were conducted using SurveyMonkey (www.surveymonkey.com), non‐FPs answered a web questionnaire on the X company platform.

### 2.4 Measures

#### 2.4.1 Dependent variable: Tolerance for ambiguity specific to the clinical context

Tolerance for ambiguity in the clinical context was measured using the Japanese version of the Tolerance for Ambiguity in Medical Students and Doctors (J‐TAMSAD) scale.^14^, ^30^ This scale has good reliability and validity.^30^ It has 18 items, each of which is answered on a 5‐point Likert scale as 1 = strongly disagree to 5 = strongly agree. Factor analysis showed a five‐factor structure: “likes complicated, challenging, and vague situations in medical practice” (Factor 1; six items); “likes the mystery that is medicine” (Factor 2; three items); “tolerance for medical settings without a single solution” (Factor 3; two items); “tolerance for things that are not black or white in medicine” (Factor 4; three items); and “tolerance for controversial circumstances in medical practice” (Factor 5; five items).^30^ The following formula was used to calculate the scale scores: J‐TAMSAD total score = 25 × (mean score of the 18 items − 1). Factor score was calculated by the following formula: Factor Y score = 25 × (mean score of the items of Factor Y − 1). The scale scores and factor scores range from 0 to 100, with higher scores indicating a greater ambiguity tolerance.^30^

#### 2.4.2 Independent variable: Specialty

The specialty of the participants was treated as a dummy variable. We classified specialty into the following categories: family medicine (reference category), internal medicine or pediatrics, surgical medicine, and other departments. The surgeon group was composed of physicians practicing in various surgical fields, including orthopedic surgery, general surgery, urology, neurosurgery, otorhinolaryngology, ophthalmology, obstetrics and gynecology, and plastic surgery. Physicians with other specialties consisted of those practicing in specialties such as psychiatry, dermatology, anesthesiology, radiology, emergency medicine, rehabilitation medicine, and others.

#### 2.4.3 Covariates

We collected data regarding participants' sociodemographic information to control for possible confounding variables (covariates). We included covariates for PGYs (3–6 (reference category); 7–15; 16–25; or ≥26) and gender (female; male (reference category); or other) with reference to previous research.^14^, ^20^, ^30^ These covariates were treated as categorical variables.

### 2.5 Statistical analysis

Descriptive statistics for participants' characteristics were computed. To examine whether specialty was associated with ambiguity tolerance in the clinical context, we used multivariable linear regression analysis. Assumptions of residual normality and equal variance were checked to ensure that they were met. Statistical significance was set as a 2‐sided *p* < 0.05.

Previous research on sample size formula indicated that a sample size per independent variable value of ≥20 was required for linear regression analysis.^31^ Accordingly, since the number of independent variables was eight, we estimated a minimum sample size of 160. All statistical analyses were performed using SPSS Statistics version 29.0 (IBM Corp).

### 2.6 Ethical considerations

In this study, at the beginning of the online questionnaire, participants were asked to read a research document explaining data anonymization, voluntary participation, and absence of disadvantage for not participating in the study. Only participants who showed a willingness to participate in the study by checking the consent box were included in the study. The study was approved by the ethics committee of Keio University School of Medicine (approval number: 20231161).

## 3 RESULTS

Of the eligible participants, 388 completed the questionnaire. For FPs, of the 651 eligible participants, 178 (27.3%) answered the questionnaire. Of the non‐FPs, 210 completed the survey although the response rate is unknown due to our survey design (i.e., web surveys). There were no missing data.

Participants' specialties are shown in Table 1, and other characteristics of the participants are shown in Table 2. The FP group had a greater proportion of female. We noted a trend indicating that FPs had higher J‐TAMSAD scale scores than non‐FPs.

**TABLE 1:** Participants' specialties (*N* = 388).

Specialty	*N* (%)
Family medicine	178 (45.9)
Internal medicine	32 (8.2)
Orthopedic surgery	28 (7.2)
Surgery	24 (6.2)
Psychiatry	20 (5.2)
Pediatrics	19 (4.9)
Urology	14 (3.6)
Neurosurgery	12 (3.1)
Dermatology	11 (2.8)
Otorhinolaryngology	10 (2.6)
Anesthesiology	9 (2.3)
Ophthalmology	9 (2.3)
Radiology	6 (1.5)
Emergency medicine	5 (1.3)
Obstetrics and gynecology	4 (1.0)
Plastic surgery	4 (1.0)
Rehabilitation	2 (0.5)
Others	1 (0.3)

**TABLE 2:** Characteristics of the participants.

Characteristic	Total (*N* = 388)	FPs (*N* = 178)	Internists or pediatricians (*N* = 51)	Surgeons^a^ (*N* = 105)	Other physicians^b^ (*N* = 54)
Gender, *n* (%)					
Female	83 (21.4)	61 (34.3)	4 (7.8)	11 (10.5)	7 (13.0)
Male	304 (78.4)	117 (65.7)	47 (92.2)	94 (89.5)	46 (85.2)
Others	1 (0.3)	NA	NA	NA	1 (1.9)
PGY, *n* (%)					
3–6	71 (18.3)	56 (31.5)	5 (9.8)	5 (4.8)	5 (9.3)
7–15	119 (30.7)	63 (35.4)	21 (41.2)	20 (19.0)	15 (27.8)
16–25	101 (26.0)	41 (23.0)	11 (21.6)	34 (32.4)	15 (27.8)
>25	97 (25.0)	18 (10.1)	14 (27.5)	46 (43.8)	19 (35.2)
J‐TAMSAD scale, mean (SD)^c^					
Total	52.7 (9.7)	56.4 (10.5)	50.1 (7.8)	49.6 (7.2)	49.1 (8.2)
F1	55.5 (15.1)	59.5 (16.0)	55.2 (13.9)	51.3 (13.4)	50.5 (13.0)
F2	52.9 (17.4)	54.8 (18.3)	52.3 (16.7)	51.9 (15.9)	49.5 (17.8)
F3	35.1 (19.2)	37.7 (22.6)	29.9 (12.0)	34.2 (15.7)	32.9 (17.2)
F4	77.2 (17.5)	84.7 (13.7)	70.9 (17.7)	69.9 (17.0)	72.7 (19.9)
F5	38.8 (13.3)	40.9 (13.7)	35.3 (13.0)	37.7 (11.4)	37.3 (14.9)

*Note*: Factor 1 = Likes complicated, challenging, and vague situations in medical practice; Factor 2 = Likes the mystery that is medicine; Factor 3 = Tolerance for medical settings without a single solution; Factor 4 = Tolerance for things that are not black or white in medicine; Factor 5 = Tolerance for controversial circumstances in medical practice.

Abbreviations: F, factor; FP, family physician; J‐TAMSAD, Japanese version of the Tolerance of Ambiguity in Medical Students and Doctors; NA, not applicable; PGY, postgraduate year; SD, standard deviation.

^a^ Physicians practicing in orthopedic surgery, general surgery, urology, neurosurgery, otorhinolaryngology, ophthalmology, obstetrics and gynecology, and plastic surgery.

^b^ Physicians practicing in psychiatry, dermatology, anesthesiology, radiology, emergency medicine, rehabilitation medicine, and others.

^c^ J‐TAMSAD scale scores and factor scores range from 0 to 100, with higher scores indicating greater tolerance for ambiguity in the clinical context.

Table 3 shows the association between specialty and J‐TAMSAD scale. After adjusting for gender and postgraduate years, non‐FPs had significantly lower ambiguity tolerance than FPs (internists or pediatricians: adjusted mean difference −8.17, 95% confidence interval (CI) −11.06 to −5.27; surgeons: adjusted mean difference −8.77, 95% CI −11.16 to −6.38; other physicians: adjusted mean difference −8.96, 95% CI −11.82 to −6.10). Non‐FPs had lower tolerance for ambiguity in all dimensions other than “Likes the mystery that is medicine,” after controlling for possible confounders. The dimension scores of “Likes the mystery that is medicine” were lower in surgeons and other physicians than in FPs, after adjustment for possible confounding factors.

**TABLE 3:** Differences in J‐TAMSAD scale scores between FPs and non‐FPs (*N* = 388).

	Unadjusted mean difference (95% CI) in score	Adjusted^a^ mean difference (95% CI) in score
J‐TAMSAD scale^b^		
Total score		
FPs	Ref.	Ref.
Internists or pediatricians	−6.25 (−9.09 to −3.41)**	−8.17 (−11.06 to −5.27)**
Surgeons	−6.78 (−8.98 to −4.59)**	−8.77 (−11.16 to −6.38)**
Other physicians	−7.23 (−10.01 to −4.46)**	−8.96 (−11.82 to −6.10)**
F1		
FPs	Ref.	Ref.
Internists or pediatricians	−4.28 (−8.86 to 0.31)	−5.53 (−10.30 to −0.75)*
Surgeons	−8.19 (−11.74 to −4.65)**	−9.02 (−12.97 to −5.08)**
Other physicians	−9.04 (−13.52 to −4.56)**	−9.86 (−14.57 to −5.14)**
F2		
FPs	Ref.	Ref.
Internists or pediatricians	−2.54 (−7.96 to 2.89)	−4.29 (−9.94 to 1.37)
Surgeons	−3.00 (−7.20 to 1.21)	−4.82 (−9.49 to −0.14)*
Other physicians	−5.29 (−10.59 to 0.02)	−6.86 (−12.44 to −1.27)*
F3		
FPs	Ref.	Ref.
Internists or pediatricians	−7.81 (−13.75 to −1.87)*	−11.42 (−17.47 to −5.36)**
Surgeons	−3.54 (−8.15 to 1.06)	−8.42 (−13.42 to −3.42)**
Other physicians	−4.84 (−10.65 to 0.97)	−9.11 (−15.08 to −3.13)**
F4		
FPs	Ref.	Ref.
Internists or pediatricians	−13.78 (−18.81 to −8.74)**	−15.45 (−20.69 to −10.22)**
Surgeons	−14.77 (−18.67 to −10.87)**	−16.22 (−20.54 to −11.89)**
Other physicians	−12.01 (−16.93 to −7.08)**	−12.88 (−18.05 to −7.72)**
F5		
FPs	Ref.	Ref.
Internists or pediatricians	−5.58 (−9.70 to −1.46)**	−7.95 (−12.18 to −3.72)**
Surgeons	−3.13 (−6.32 to 0.06)	−5.95 (−9.45 to −2.45)**
Other physicians	−3.60 (−7.63 to 0.43)	−6.18 (−10.36 to −2.00)**

*Note*: Factor 1 = Likes complicated, challenging, and vague situations in medical practice; Factor 2 = Likes the mystery that is medicine; Factor 3 = Tolerance for medical settings without a single solution; Factor 4 = Tolerance for things that are not black or white in medicine; Factor 5 = Tolerance for controversial circumstances in medical practice.

Abbreviations: CI, confidence interval; F, factor; FP, family physician; J‐TAMSAD, Japanese version of the Tolerance of Ambiguity in Medical Students and Doctors.

^a^ Adjusted for gender and postgraduate years.

^b^ J‐TAMSAD scale scores and factor scores range from 0 to 100, with higher scores indicating greater tolerance for ambiguity in the clinical context.

* *p* < 0.05.

** *p* < 0.01.

## 4 DISCUSSION

This study investigated differences in ambiguity tolerance specific to the clinical context between FPs and non‐FPs. The results of the multivariable linear regression analysis revealed that FPs had significantly greater clinical context‐specific ambiguity tolerance than non‐FPs. Our findings suggest that there may be a need to improve ambiguity tolerance among non‐FPs through educational intervention, as these clinicians are increasingly involved in ambiguous situations in today's medicine, regardless of specialty.^3^ FPs and non‐FPs should work together to complement each other's strengths, rather than simply improving the training for non‐FPs.

Our findings are inconsistent with those of a US study by Geller et al. in the early 1990s.^20^ As noted above, these authors showed that the ambiguity tolerance of family practitioners was rather lower than that of physicians in other specialties. We suggest four possible reasons to explain this discrepancy. First, the type of ambiguity tolerance measured differed between our present and this previous paper. Geller et al.^20^ measured general ambiguity tolerance as a personality trait. Conversely, we used the scale to assess clinical context‐specific tolerance for ambiguity. Second, between the early 1990s and today, the quality of ambiguity in the clinical context may have changed. The recent availability of clinical guidelines, medical search engines,^32^ and medical applications for mobile devices^33^ have affected the scope and nature of ambiguity in medical practice. Conversely, complexity and diversity in clinical settings continue to grow,^34^ leading physicians to face a different kind of ambiguity than before in clinical decision making (e.g., increasing diversity of patients and the growing number of patients with multimorbidity).^35^, ^36^ Third, there are differences in practice style between family practitioners in the United States and FPs in Japan. FPs in Japan work flexibly according to the needs of their respective practice sites. Some provide inpatient, outpatient, and home‐visit care. Fourth, there may be cultural differences in attitudes towards ambiguity between the United States and Japan. The cultural differences between the United States and Japan are likely to have an influence on the responses to the questionnaire.

The mechanism by which FPs have a greater tolerance for ambiguity may be explained by the following two points. First, medical trainees with more ambiguity tolerance may choose family medicine as a specialty. However, this hypothesis is questionable given that several recent studies did not identify a significant association between ambiguity tolerance and specialty preference among medical trainees.^14^, ^23^ Second, FPs develop their ambiguity tolerance as they gain experience. As noted in the Introduction, FPs often work in a clinical environment filled with ambiguity (e.g., patients with multimorbidity, patients with undifferentiated problems, and limited access to testing), which may lead to the development of ambiguity tolerance. Alternatively, reflection with an experienced supervisor may foster a trainee's tolerance for ambiguity. Further study is required to clarify the mechanism.

To our knowledge, this is the first study to investigate differences in tolerance for ambiguity specific to the clinical context between FPs and non‐FPs. Our findings are based on data from a nationwide survey. The measure used in the study, the TAMSAD scale, is well validated and used worldwide in the field of medical education. Thus, the findings of the present study have relatively good validity. On the other hand, Japan has a unique healthcare system that guarantees free access (i.e., patients are free to choose when and where they see a physician, regardless of the size of the facility or the specialty) as well as a rapidly aging population and increasing number of patients with multimorbidity.^37^, ^38^ In addition, FPs are sometimes required to provide not only outpatient care, but also inpatient and home care. This unique context of the Japanese healthcare system may increase the ambiguity tolerance of Japanese FPs particularly strongly. Further insight into ambiguity tolerance may be gained by conducting similar studies internationally and comparing the results with those of our present study in Japan.

There are some potential limitations in the study. First, our study was conducted under a cross‐sectional design, and accordingly does not allow any determination of the causal directions between specialty and tolerance for ambiguity in the clinical context. Second, although we determined covariates with reference to previous research, unknown confounding factors may still be present. Third, the response rate was a concern; physicians with lower ambiguity tolerance were less likely to complete our survey. Fourth, the possibility of information bias cannot be excluded. However, we used a number of methods to minimize this concern (e.g., anonymous survey design). Fifth, although this study indicated statistical significance, the clinical and educational relevance of the score difference remains unknown. Future research to examine the interpretability of the J‐TAMSAD scale may clarify the significance of our study.

## 5 CONCLUSIONS

We investigated differences in tolerance of ambiguity in the clinical context between FPs and non‐FPs. The results of our multivariable linear regression analysis showed that FPs had significantly greater tolerance for ambiguity specific to the clinical context. Our findings suggest that there may be a need to improve the non‐FPs' ambiguity tolerance in the clinical context through educational interventions, given that today's medicine provides increased exposure to ambiguity, regardless of specialty.

## FUNDING INFORMATION

This work was supported by the Pfizer Health Research Foundation, Japan (grant no. 23‐Y‐13) and the Japan Society for the Promotion of Science, Japan (grant no. JP23K19809).

## CONFLICT OF INTEREST STATEMENT

The authors have stated explicitly that there are no conflicts of interest in connection with this article.

## ETHICS STATEMENT

Ethical approval statement: Ethical approval was granted by the ethical committee of Keio University School of Medicine on December 28, 2023 (approval number: 20231161).

Patient consent statement: At the beginning of the questionnaire, all participants were asked to read a description of the study and check the consent box to participate in the study.

Clinical trial registration: None.

## Data Availability

Upon reasonable request, the corresponding author can provide the data sets generated and analyzed in the study.

## References

[1] Luther VP , Crandall SJ . Commentary: ambiguity and uncertainty: neglected elements of medical education curricula? Acad Med. 2011;86(7):799–800. 21715991 10.1097/ACM.0b013e31821da915

[2] Geller G , Grbic D , Andolsek KM , Caulfield M , Roskovensky L . Tolerance for ambiguity among medical students: patterns of change during medical school and their implications for professional development. Acad Med. 2021;96(7):1036–1042. 33149092 10.1097/ACM.0000000000003820

[3] Maini A , Saravanan Y , Singh TA , Fyfe M . Coaching skills for medical education in a VUCA world. Med Teach. 2020;42(11):1308–1309. 32657666 10.1080/0142159X.2020.1788713

[4] Hillen MA , Gutheil CM , Strout TD , Smets EMA , Han PKJ . Tolerance of uncertainty: conceptual analysis, integrative model, and implications for healthcare. Soc Sci Med. 2017;180(1):62–75. 28324792 10.1016/j.socscimed.2017.03.024

[5] Wayne S , Dellmore D , Serna L , Jerabek R , Timm C , Kalishman S . The association between intolerance of ambiguity and decline in medical students' attitudes toward the underserved. Acad Med. 2011;86(7):877–882. 21617507 10.1097/ACM.0b013e31821dac01

[6] Raglan GB , Babush M , Farrow VA , Kruglanski AW , Schulkin J . Need to know: the need for cognitive closure impacts the clinical practice of obstetrician/gynecologists. BMC Med Inform Decis Mak. 2014;14(1):122. 25540033 10.1186/s12911-014-0122-6 PMC4297425

[7] Nevalainen M , Kuikka L , Sjoberg L , Eriksson J , Pitkala K . Tolerance of uncertainty and fears of making mistakes among fifth‐year medical students. Fam Med. 2012;44(4):240–246. 22481152

[8] Carney PA , Yi JP , Abraham LA , Miglioretti DL , Aiello EJ , Gerrity MS , et al. Reactions to uncertainty and the accuracy of diagnostic mammography. J Gen Intern Med. 2007;22(2):234–241. 17356992 10.1007/s11606-006-0036-9 PMC1824735

[9] Ghosh AK . On the challenges of using evidence‐based information: the role of clinical uncertainty. J Lab Clin Med. 2004;144(2):60–64. 15322499 10.1016/j.lab.2004.05.013

[10] Benbassat J , Pilpel D , Schor R . Physicians' attitudes toward litigation and defensive practice: development of a scale. Behav Med. 2001;27(2):52–60. 11763825 10.1080/08964280109595771

[11] Fujikawa H , Aoki T , Son D , Hayashi M , Eto M . Association between tolerance for ambiguity specific to the clinical context and empathy in medical trainees: a multicenter cross‐sectional study in Japan. Med Teach. 2024;46(4):512–518. 37734453 10.1080/0142159X.2023.2259065

[12] Geller G , Faden RR , Levine DM . Tolerance for ambiguity among medical students: implications for their selection, training and practice. Soc Sci Med. 1990;31(5):619–624. 2218644 10.1016/0277-9536(90)90098-d

[13] Begin AS , Hidrue M , Lehrhoff S , del Carmen MG , Armstrong K , Wasfy JH . Factors associated with physician tolerance of uncertainty: an observational study. J Gen Intern Med. 2021;37(6):1415–1421. 33904030 10.1007/s11606-021-06776-8 PMC8074695

[14] Hancock J , Roberts M , Monrouxe L , Mattick K . Medical student and junior doctors' tolerance of ambiguity: development of a new scale. Adv Health Sci Educ Theory Pract. 2015;20(1):113–130. 24841480 10.1007/s10459-014-9510-z

[15] Leung J , Cloninger CR , Hong BA , Cloninger KM , Eley DS . Temperament and character profiles of medical students associated with tolerance of ambiguity and perfectionism. PeerJ. 2019;7(1):e7109. 31223537 10.7717/peerj.7109 PMC6571128

[16] Lodewyk K , Linkiewich D , Lee A , Babenko O . From jerseys to scrubs: is sport background associated with medical students' tolerance of ambiguity and uncertainty? Health Prof Educ. 2020;6(4):501–505.

[17] O'Riordan M , Dahinden A , Akturk Z , Ortiz JM , Dağdeviren N , Elwyn G , et al. Dealing with uncertainty in general practice: an essential skill for the general practitioner. Qual Prim Care. 2011;19(3):175–181. 21781433

[18] Turner N . Family physicians: first point of contact, last line of defence. Can Fam Physician. 2023;69(7):490–491. 37451998 10.46747/cfp.6907490 PMC10348802

[19] Katerndahl D , Wood R , Jaen CR . Family medicine outpatient encounters are more complex than those of cardiology and psychiatry. J Am Board Fam Med. 2011;24(1):6–15. 21209339 10.3122/jabfm.2011.01.100057

[20] Geller G , Tambor ES , Chase GA , Holtzman NA . Measuring physicians' tolerance for ambiguity and its relationship to their reported practices regarding genetic testing. Med Care. 1993;31(11):989–1001. 8231339 10.1097/00005650-199311000-00002

[21] Bovier PA , Perneger TV . Stress from uncertainty from graduation to retirement—a population‐based study of Swiss physicians. J Gen Intern Med. 2007;22(5):632–638. 17443371 10.1007/s11606-007-0159-7 PMC1855273

[22] Levaillant M , Levaillant L , Lerolle N , Vallet B , Hamel‐Broza J‐F . Factors influencing medical students' choice of specialization: a gender based systematic review. EClinicalMedicine. 2020;28(1):100589. 33134904 10.1016/j.eclinm.2020.100589 PMC7588859

[23] Babenko O , Linkiewich D , Lodewyk K , Lee A . Ambiguity tolerance and prospective specialty choice among third‐year medical students. PRiMER. 2021;5(1):2. 33860157 10.22454/PRiMER.2021.453158 PMC8041222

[24] Yamamoto Y , Haruta J , Goto R , Maeno T . What kinds of work do Japanese primary care physicians who derive greater positive meaning from work engage in? A cross‐sectional study. J Gen Fam Med. 2023;24(2):94–101. 36909785 10.1002/jgf2.595 PMC10000278

[25] Yokota Y , Watari T . Various perspectives of “general medicine” in Japan—respect for and cooperation with each other as the same “general medicine physicians”. J Gen Fam Med. 2021;22(6):314–315. 34754709 10.1002/jgf2.500 PMC8561093

[26] Japan Primary Care Association . About the association. 2024. [cited 2024 Mar 8]. Available from: https://www.primarycare‐japan.com/about.htm

[27] WONCA . WONCA Accreditation and Certification. 2021. [cited 2024 Mar 8]. Available from: https://www.globalfamilydoctor.com/News/WONCAAccreditationandCertification.aspx

[28] The Japanese Medical Specialty Board . About The Japanese Medical Specialty Board. 2024. [cited 2024 Mar 8]. Available from: https://jbgm.org/

[29] Kato D , Ryu H , Matsumoto T , Abe K , Kaneko M , Ko M , et al. Building primary care in Japan: literature review. J Gen Fam Med. 2019;20(5):170–179. 31516802 10.1002/jgf2.252 PMC6732569

[30] Fujikawa H , Son D , Hayashi M , Kondo K , Eto M . Translation, adaptation, and validation of the tolerance of ambiguity in medical students and doctors (TAMSAD) scale for use in Japan. BMC Med Educ. 2023;23(1):405. 37277759 10.1186/s12909-023-04391-1 PMC10240119

[31] Hair JF , Black WC , Babin BJ , Anderson RE . Multivariate data analysis. 8th ed. Andover, MA: Cengage Learning EMEA; 2019.

[32] Labrecque M , Ratte S , Fremont P , Cauchon M , Ouellet J , Hogg W , et al. Decision making in family medicine: randomized trial of the effects of the InfoClinique and trip database search engines. Can Fam Physician. 2013;59(10):1084–1094. 24130286 PMC3796978

[33] Lee M , Bin Mahmood ABS , Lee ES , Smith HE , Tudor CL . Smartphone and mobile app use among physicians in clinical practice: scoping review. JMIR Mhealth Uhealth. 2023;11(1):e44765. 37000498 10.2196/44765 PMC10131676

[34] Verbree A‐R , Isik U , Janssen J , Dilaver G . Inclusion and diversity within medical education: a focus group study of students' experiences. BMC Med Educ. 2023;23(1):61. 36698110 10.1186/s12909-023-04036-3 PMC9875758

[35] Chowdhury SR , Chandra Das D , Sunna TC , Beyene J , Hossain A . Global and regional prevalence of multimorbidity in the adult population in community settings: a systematic review and meta‐analysis. EClinicalMedicine. 2023;57(1):101860. 36864977 10.1016/j.eclinm.2023.101860 PMC9971315

[36] Dharamsi S , Ho A , Spadafora SM , Woollard R . The physician as health advocate: translating the quest for social responsibility into medical education and practice. Acad Med. 2011;86(9):1108–1113. 21785306 10.1097/ACM.0b013e318226b43b

[37] Aoki T , Yamamoto Y , Ikenoue T , Onishi Y , Fukuhara S . Multimorbidity patterns in relation to polypharmacy and dosage frequency: a nationwide, cross‐sectional study in a Japanese population. Sci Rep. 2018;8(1):3806. 29491441 10.1038/s41598-018-21917-6 PMC5830504

[38] Sakamoto H , Rahman MM , Nomura S , Okamoto E , Koike S , Yasunaga H , et al. Japan health system review. 2018. [cited 2024 Mar 8]. Available from: https://apps.who.int/iris/bitstream/handle/10665/259941/9789290226260‐eng.pdf?sequence=1&isAllowed=y

